# Stem cells from human exfoliated deciduous teeth ameliorate type II diabetic mellitus in Goto-Kakizaki rats

**DOI:** 10.1186/s13098-019-0417-y

**Published:** 2019-02-28

**Authors:** Nanquan Rao, Xiaotong Wang, Yue Zhai, Jingzhi Li, Jing Xie, Yuming Zhao, Lihong Ge

**Affiliations:** 1 Department of Pediatric Dentistry, Peking University School and Hospital of Stomatology, 22 Zhongguancun Avenue South, Haidian District, Beijing, 100081 People’s Republic of China; 2 Department of Oral Emergency Department, Peking University School and Hospital of Stomatology, 22 Zhongguancun Avenue South, Haidian District, Beijing, 100081 People’s Republic of China; 3 Department of Stomatology, Shenzhen Children’s Hospital, No. 7019, Yitian Road, Shenzhen, 518026 People’s Republic of China

**Keywords:** Stem cells from human exfoliated deciduous teeth (SHED), Type 2 diabetes mellitus, Goto-Kakizaki (GK) rats, Insulin resistance

## Abstract

**Background:**

By 2030, diabetes mellitus (DM) will be the 7th leading cause of death worldwide. Type 2 DM (T2DM) is the most common type of DM and is characterized by insulin resistance and defective β-cell secretory function. Stem cells from human exfoliated deciduous teeth (SHED) are favorable seed cells for mesenchymal stem cells (MSCs)-based therapy due to their higher proliferation rates and easier access to retrieval. Currently, no study has revealed the therapeutic efficiency of MSCs for T2DM in Goto-Kakizaki (GK) rats. Hence, we aimed to explore the effect of SHED on T2DM in GK rats.

**Materials and methods:**

We investigated the effects of SHED on the progression of T2DM in GK rats. SHED and bone marrow mesenchymal stem cells (BMSCs) were injected via the tail vein. Body weight, fasting blood glucose and non-fasting blood glucose were measured before and after administration. At 8 weeks after injection, intraperitoneal insulin tolerance tests (IPITTs) and insulin release tests (IRTs) were performed. Additionally, hematoxylin–eosin (HE) staining, periodic acid-Schiff (PAS) staining and double-label immunofluorescence staining were used to explore the pathological changes in pancreatic islets and the liver. Immunohistochemistry (IHC) was employed to detect SHED engraftment in the liver. Additionally, real-time PCR and western blotting were used to explore glycogen synthesis, glycolysis and gluconeogenesis in the liver.

**Results:**

At 8 weeks after SHED injection, T2DM was dramatically attenuated, including hyperglycemia, IPGTT and IRT. Additionally, histological analysis showed that SHED injection improved pancreatic islet and liver damage. Real-time PCR analysis showed that SHED significantly reversed the diabetic-induced increase of G-6-Pase, Pck1 and PK; and significantly reversed the diabetic-induced decrease of GSK3β, GLUT2, and PFKL. In addition, western blotting demonstrated that SHED significantly reversed the diabetic-induced increase of G-6-Pase and reversed the diabetic-induced decrease of GLUT2, GSK3β and PFKM.

**Conclusion:**

Stem cells from human exfoliated deciduous teeth offers a potentially effective therapeutic modality for ameliorating T2DM, including hyperglycemia, insulin resistance, pancreatic islets and liver damage, and decreased glycogen synthesis, inhibited glycolysis and increased gluconeogenesis in the liver.

## Background

Currently, though artificial synthesis and extensive application of insulin have substantially decreased the mortality associated with DM and improved the quality of life of DM patients and related complications, more than 400 million people throughout the world with DM continue to suffer from devastating secondary complications [1]. T2DM is the most common type of DM and is characterized by progressively inexorable β-cell dysfunction and insulin resistance in skeletal muscle, adipose tissue, and the liver [2]. In addition, currently available therapeutic regimens either target insulin resistance or insulin deficiency [3]. Excellent metabolic control without the need for exogenous insulin can be achieved with pancreas transplantation or pancreatic islet transplantation. While the procedure is associated with adverse effects in a limited number of available donors, immunosuppressive regimens [4] have immunological risks that affect long-term survival [5].

Therefore, MSCs appear to be an ideal tool for treating DM and the related secondary complications as the cells can be easily isolated from bone marrow, adipose tissue, cord blood and dental pulp and can be rapidly expanded in vitro [6]. Importantly, MSCs are hypoimmunogenic. When systemically administered, MSCs home to injured organs, contribute to tissue regeneration and have been transplanted into human patients with different diseases with beneficial effects and without major toxicity [7–9]. The antidiabetic effect of autologous and allogeneic MSCs has been demonstrated in different animal models of and patients with T1DM [10–12] and T2DM [13, 14]. It is widely accepted that MSCs might contribute to tissue regeneration due to their ability to regulate the local microenvironment by paracrine mechanisms [15–17]. MSCs limit the expansion and cytotoxic activity of T lymphocytes and stimulate the appearance of regulatory T cells [18]. Furthermore, MSCs secrete anti-inflammatory cytokines and inhibit the expression of pro-inflammatory cytokines by immune cells [19, 20]. Moreover, MSCs are able to produce both in vitro and in vivo anti-apoptotic and mitogenic factors, including epidermal growth factor (EGF), hepatocyte growth factor (HGF), insulin-like growth factor-1 (IGF1), and basic fibroblast growth factor (bFGF). The biological effects of these trophic factors can be direct (triggering intracellular signaling) or indirect (inducing neighboring cells to secrete other bioactive factors) [21, 22].

Miura et al. [23] found that SHED have superior proliferative capacity, self-renewal ability, and multidirectional differentiation potential. SHED are MSCs derived from dental pulps in exfoliated deciduous teeth from young patients. In addition, SHED have immunomodulatory abilities [24]. SHED have a good prospect in the treatment of T1DM [25], liver fibrosis [26], lupus erythematosus [27], and spinal cord injury [28].

Therefore, we hypothesize that SHED could be beneficial for the progression of T2DM. In this study, GK rats, a non-obese and spontaneous (genetic) T2DM model, was first used to examine the effect of MSCs on T2DM.

## Materials and methods

### SHED isolation, in vitro expansion and characterization

The SHED donors were patients aged 6 to 8 years old from a pediatric clinic. The protocol was approved by the Ethics Committee (PKUSSIRB-201630091). The isolation of SHED was performed following Miura et al’s reports [22]. Briefly, the pulp tissues were collected from deciduous teeth, cut into small pieces, then digested with 0.1% type I collagenase (Sigma-Aldrich, St. Louis, MO, USA) and dispase (Sigma-Aldrich, St. Louis, MO, USA) in α-MEM (Hyclone, Logan, UT, USA) at 37 °C for 60 min. Then, α-MEM containing 20% FBS (Gibco, Mulgrave, VIC, Australia) was added and subsequently centrifuged at 1000 rpm/min for 5 min. Next, the supernatant was discarded and the cell pellet was resuspended and cultured at room temperature in α-MEM containing 20% FBS, 100 U/mL penicillin and 100 μg/mL streptomycin (Solarbio, Beijing, China) at 37 °C in 5% CO_2_ for 3 days. The culture solution was changed every 3 or 4 days.

Human BMSCs were purchased from ScienCell (San Diego, California, USA) and cultured in α-MEM containing 10% FBS, 100 U/mL penicillin and 100 μg/mL streptomycin.

When monolayer SHED and BMSCs confluence was observed, the cells were passaged at a ratio of 1:3 and cultured with α-MEM supplemented with 10% FBS and 1% antibiotics at 37 °C in 5% CO_2_. Cells from passage 3 to 4 were used in the present study.

The surface marker profiles of SHED were tested with flow cytometry. The third passage of cells was resuspended in cold PBS containing 2% FBS at a concentration of 1 × 10^6^ cells/mL prior to adding the following monoclonal antibodies: CD34-PE, CD45-PE, CD73-PE, CD90-FITC, CD105-FITC and CD146-PE (Beckman Coulter, Brea, CA, USA). The unmarked cells were used as a negative control. Finally, the stained cells were analyzed using a Beckman Coulter flow cytometry system (FC500, Beckman Coulter, Brea, CA, USA).

### Adipogenic and osteogenic differentiation

To induce adipogenic differentiation, confluent adherent cells were cultured in α-MEM containing 10% FBS, 100 U/mL penicillin and 100 μg/mL streptomycin, supplemented with 1 μM dexamethasone (Sigma-Aldrich, St Louis, MO, USA), 100 μg/mL 3-isobutyl-1-methyxathines (Sigma-Aldrich, St Louis, MO, USA), 100 μM indomethacin (Sigma-Aldrich, St Louis, MO, USA) and 200 μM/mL insulin (Sigma-Aldrich, St Louis, MO, USA), replaced every 2 days. After 21 days of stimulation, cell differentiation into lipid-laden adipocytes was confirmed by Oil Red O (Sigma-Aldrich, St. Louis, MO, USA) staining.

To induce osteogenic differentiation, confluent adherent cells were cultured in media containing 10% FBS and 100 U/mL penicillin and 100 μg/mL streptomycin, supplemented with 0.1 μM dexamethasone, 10 mM β-glycerophosphate (Sigma-Aldrich, St. Louis, MO, USA) and 50 μg/mL ascorbate 2-phosphate (Sigma-Aldrich, St. Louis, MO, USA), replaced every 2 days. After 14 days of stimulation, cells that differentiated into hydroxyapatite-producing osteoblasts were confirmed by Alizarin Red (Sigma-Aldrich, St. Louis, MO, USA) staining.

### Animal model and groups

Twenty-five SPF grade GK rats (12 weeks old, male) were purchased from Changzhou Cavens Laboratory Animal Co., Ltd (Changzhou, Jiangsu, China). Eight SPF grade Wistar rats (12 weeks old, male) were purchased from Beijing Vital River Laboratory Animal Technology Co., Ltd (Beijing, China) as normal controls. The rats were maintained on a 12 h light:12 h dark cycle with free access to rodent chow and water. All animal experiments were conducted in accordance with the protocols and guidelines of the PU IRB Laboratory Animal Welfare Committee.

After GK rats consumed a high fat diet for 4 weeks, rats with a non-fasting blood glucose ≥ 11.1 mM for 3 consecutive days were classified as T2DM rats. T2DM rats were randomly divided into three groups and eight normal Wistar rats were used as a normal control group. Then, T2DM rats were subsequently fed conventional chow. Rats in four groups received the following treatment: (1) normal Wistar rats without treatment (normal group, n = 8); (2) GK rats received SHED infusion (SHED group, n = 8); (3) GK rats received 1 mL of PBS infusion (PBS group, n = 8); (4) GK rats received BMSC infusion (BMSCs group, n = 8). All rats in the four groups were observed for 8 weeks after administration.

In total, 4 × 10^6^ cells, including SHED and BMSCs, were resuspended in 1 mL PBS and administered via tail vein to each rat. The PBS group received 1 mL PBS.

### Physical and biochemical assessment

Body weight, fasting blood glucose and non-fasting blood glucose were measured each week.

At 8 weeks after administration, IPITTs and IRTs were performed. Plasma glucose levels were monitored throughout the experiments with ACCU-CHEK Performa (Roche Diagnostic, Basel, Switzerland). Serum insulin measurement was performed by ELISA (Rat Insulin Elisa Kit; Millipore, St. Charles, MO).

Fasting blood glucose (FBG) and fasting serum insulin (FINS) concentrations were measured in blood collected through tail prick at 8 weeks after MSCs injection. The homeostatic model assessment (HOMA) described previously was used to assess changes in pancreatic β-cell function (HOMA-β) and insulin resistance (HOMA-IR) in treated groups during the experimental period (22). The following equations were used to calculate the HOMA-IR index and HOMA-β index: HOMA-IR index = (FBG [in mmol/L] 3 FINS [in units/L])/22.5 and HOMA-b = (20 * FINS [in units/L])/(FBG [in mmol/L] − 3.5).

### Pancreatic islet histological and immunohistochemical analysis

After IPGTT tests, the rats were given free access to rodent chow and water. Three days later, the rats were sacrificed using 10% chloral hydrate (Solarbio, Beijing, China). Liver tissues and pancreatic tissue were collected for further investigation.

Pancreatic tissue was embedded in paraffin. Paraffin sections (4 μm) were stained with hematoxylin–eosin reagent. The number of regular and irregular pancreatic islets was counted, and the ratio of irregular islets to total islets was calculated. Five slices from each rat were randomly collected for statistics.

For double-label immunofluorescence staining, sections were deparaffinized, rehydrated and submitted to Tris–EDTA, pH 9.0 antigen retrieval solution (Zhongshan Golden Bridge Biotechnology, Beijing, China) in a microwave for 30 min. Next, 3% hydrogen peroxide (Zhongshan Golden Bridge Biotechnology, Beijing, China) was used to block the endogenous peroxide. Then, rabbit anti-rat insulin and mouse anti-rat glucagon primary antibodies (Abcam, Cambridge, UK) were added, and the specimens were incubated at 4 °C overnight and washed thoroughly with PBS. The sections were incubated with a secondary antibody (Alexa Fluor 488-conjugated goat anti-rabbit IgG and Alexa Fluor 594–conjugated goat anti-mouse IgG, Abcam, Cambridge, UK) at room temperature for 2 h.

Subsequently, pancreatic sections were stained with DAPI (Solarbio, Beijing, China) and mounted. The sections were observed by fluorescence microscopy. α-Cells and β-cells were recorded in each section, and the ratio of β-cells to total islet cells was calculated. Five slices from each rat were randomly collected for statistics.

### Liver histological analysis and SHED detection in the liver

Liver tissue was embedded in paraffin. Liver sections (4 μm) were stained with PAS reagent (Solarbio, Beijing, China) to assess glycogen. Slices were analyzed under light microscopy, and images were captured with a digital camera.

Additionally, SHED engraftment in liver tissue was detected by immunohistochemistry in prepared sections as follows. Briefly, the sections were deparaffinized, rehydrated and submitted to Tris–EDTA, pH 9.0 antigen retrieval solution (Zhongshan Golden Bridge Biotechnology, Beijing, China) in a microwave for 30 min. Next, 3% hydrogen peroxide (Zhongshan Golden Bridge Biotechnology, Beijing, China) was used to block the endogenous peroxide. Then, the sections were incubated with human DNA PKcs primary antibody (Proteintech, Chicago, IL, USA) at 4 °C overnight. After a thorough rinse with PBS, sections were incubated with a secondary antibody using a horseradish peroxidase polymer system (Zhongshan Golden Bridge Biotechnology, Beijing, China) at room temperature for 30 min. The detection step was performed with diaminobenzidine (Zhongshan Golden Bridge Biotechnology, Beijing, China). Finally, sections were counterstained with hematoxylin to distinguish cell nuclei.

### Quantitative real-time PCR analysis and western blotting of the liver

Total RNA was isolated from liver tissues using TRIzol reagent (Invitrogen, Carlsbad, CA, USA). Then, mRNA was reverse transcribed to cDNA by using a transcriptor first-strand cDNA synthesis kit (Takara Biotechnology, Dalian, Liaoning, China). RNA expression was assessed by real time reverse transcriptase polymerase chain reaction (PCR) using a SYBR Green System (7300 Real time System, Applied Biosystem, Carlsbad, CA, USA) according to the manufacturer’s protocol. cDNA was amplified by PCR using the following primers (Table 1).

**Table 1:** Primers used for PCR amplifications

	Forward primer	Reverse primer
GADPH	5′-TGGAGTCTACTGGCGTCTT-3′	5′-TGTCATATTTCTCGTGGTTCA-3′
G6Pase	5′-AGGTGGTGGCTGGAGTCTTG-3′	5′-CTGGAGGCTGGCATTGTAGATG-3′
GLUT2	5′-CCAGCACATACGACACCAGAC-3′	5′-ACACAGACAGAGACCAGAGCATA-3′
GSK3β	5′-CGAACTCCACCAGAGGCAATC-3	5′-CGAACTCCACCAGAGGCAATC-3′
PK	5′-AACCAATATGCCTGCCTTCCAA-3′	5′-ACCAACTGCCACCTTCCACTA-3′
PFKL	5′-CCAGCCACCATCAGCAACAA-3′	5′-TGTCTGTCTTCATCTTCTCTGTCAT-3′
Pck1	5′-AACTGTTGGCTGGCTCTCACT -3′	5′-GGAACCTGGCGTTGAATGCTT-3′

Liver samples were lysed in ice-cold RIPA buffer (Solarbio, Beijing, China) with protease inhibitor cocktail (Solarbio, Beijing, China). Protein quantification was performed by BCA assay (Cwbio, Beijing, China), and equal amounts of protein lysate (50 μg) were separated by 10% SDS-PAGE. Transfer to nitrocellulose membranes was performed in transfer buffer (Millipore, Shanghai, China). After, the membranes were blocked for 2 h in TBST buffer with 5% BSA and probed with the PFKM, GSK3β, GLUT2 (Proteintech, Chicago, IL, USA), G-6-Pase (Abcam, Cambridge, UK), and β-actin antibodies (Proteintech, Chicago, IL, USA) overnight at 4 °C. The membranes were washed with TBST buffer three times, followed by incubation with the appropriate HRP conjugated secondary antibody (Proteintech, Chicago, Il, USA) for 1 h at room temperature. Finally, the protein expression levels were detected by ECL (Solarbio, Beijing, China).

### Statistical analysis

All data are presented as the mean ± standard deviation (SD). Statistical analysis was performed using SPSS software (version 17.0; SPSS Inc., Chicago, IL, USA). Student’s t test was used to compare variables before and after administration. For multiple comparisons, one-way ANOVA analysis of variance was applied. Statistical significance was set at P < 0.05.

## Results

### MSC culture and identification

**Fig. 1:**
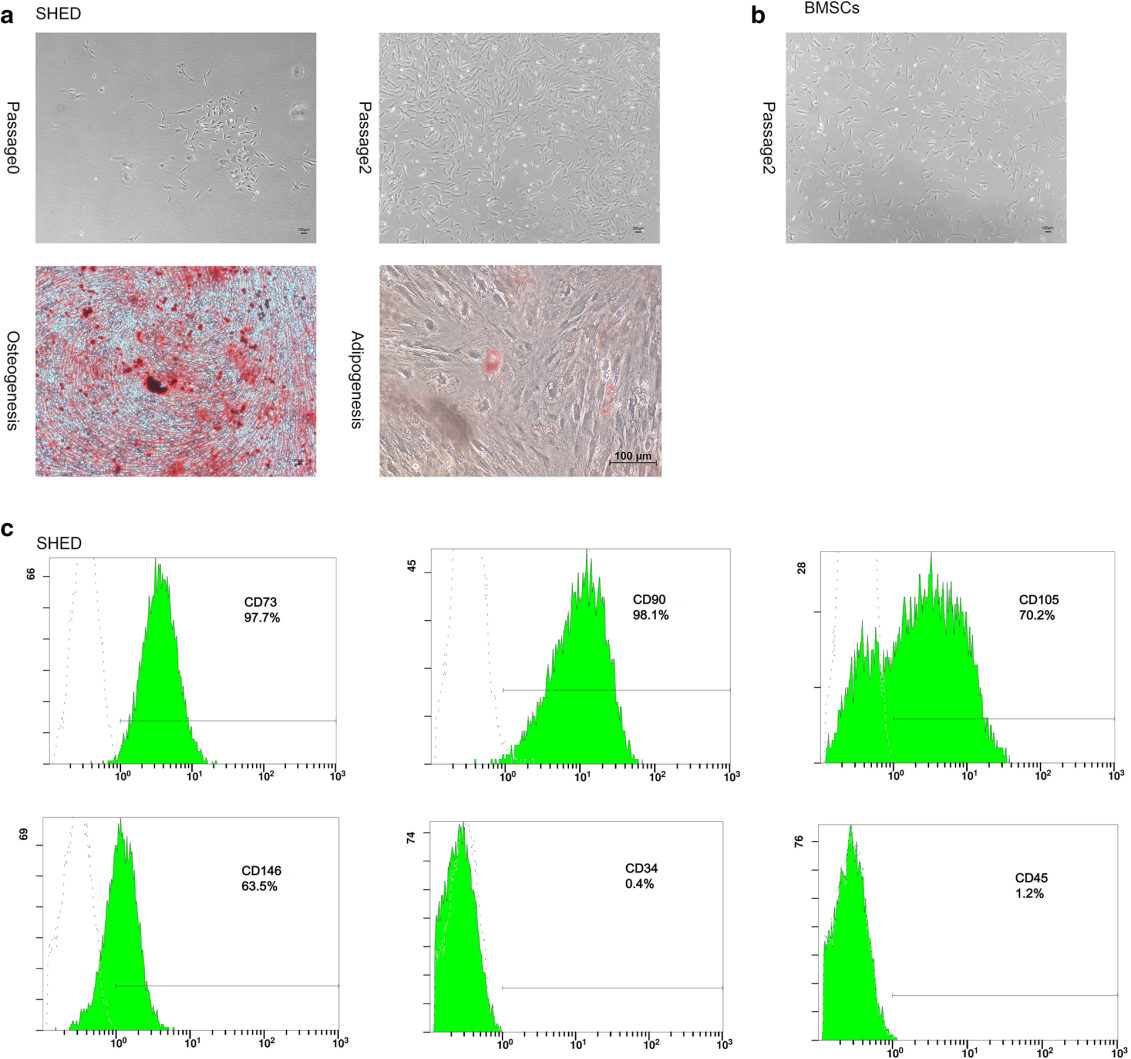
Characterization of SHED and BMSCs. **a** Characterization of SHED. SHED showed a fibroblast-like morphology. Multilineage differentiation potency including osteogenesis as identified by Alizarin Red staining; adipogenesis as identified by Oil Red O staining. **b** BMSCs showed a fibroblast-like morphology. **c** Flow cytometry of SHED. SHED expressed low levels of CD34 (0.4%) and CD45 (1.2%) and expressed high levels of CD73 (97.7%), CD90 (98.1%), CD105 (70.2%), and CD146 (63.5%)

Stem cells from human exfoliated deciduous teeth and BMSCs exhibited typical fibroblast-like morphology. SHED were differentiated into osteogenic and adipogenic cells. In addition, SHED were identified by the surface markers CD73(+), CD90(+), CD105(+), CD146(+), CD34(−) and CD45(−) by flow cytometry (Fig. 1).

### Effects of SHED on physical and biochemical parameters

Twenty-four of 25 GK rats met the condition of non-fasting blood glucose ≥ 11.1 mM for 3 consecutive days.

The weight of the rats in each group increased slowly. The body weight of the SHED group, BMSCs group and PBS group was significantly lower than that of the normal group during treatment. Within 8 weeks, fasting blood glucose and non-fasting blood glucose levels in the PBS group, BMSCs group, and SHED group were significantly higher than those in the normal group. At 1 week and 8 weeks after injection, there was no statistically significant difference in fasting glucose and non-fasting glucose among the three groups of GK rats.

At 3 weeks after injection, fasting blood glucose in the SHED group was significantly lower than in the PBS group. At 5 weeks after injection, fasting blood glucose in the SHED group and BMSCs group were significantly lower than those in the PBS group. There was not a remarkable difference between the SHED group and BMSCs group.

Meanwhile, in the PBS group, there was no significant difference in fasting blood glucose after injection compared to before; in the BMSCs group, the fasting blood glucose level decreased remarkably, lasting 6 weeks; in the SHED group, fasting blood glucose was significantly lower after injection over 8 weeks except at 5 weeks after injection.

Additionally, non-fasting blood glucose levels of T2DM rats were all significantly higher than those in the normal group during treatment. At 2, 3, 4, 6, and 7 weeks after injection, the non-fasting blood glucose level in the SHED group was significantly lower than that in the PBS group. There was no significant difference between the BMSCs group and PBS group, and there was no significant difference between the BMSCs group and SHED group. At 5 weeks after injection, non-fasting blood glucose levels in the SHED group and BMSCs group were significantly lower than in the PBS group, and there was no significant difference between the SHED group and BMSCs group.

The IPGTT test showed that blood glucose in the SHED group was significantly lower than in the PBS group at 1 h and 2 h after glucose injection.

**Fig. 2:**
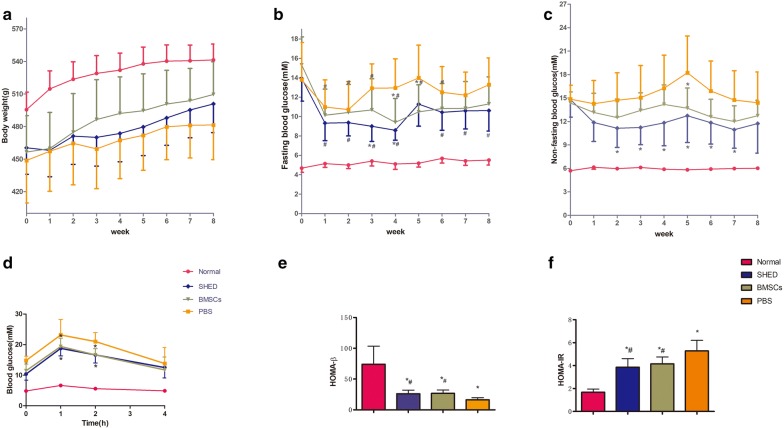
Effects of SHED on physical and biochemical parameters. **a** Body weight. **b** Fasting blood glucose. **c** Non-fasting blood glucose of rats in different groups over 8 weeks. **d** IPITTs, by injecting 2 g glucose/kg body weight. **e** HOMA-β of each group, HOMA-β (HBCI) = (20 * FINS [in units/L])/(FBG [in mmol/L] − 3.5). **f** IR index of each group, HOMA-IR index = (FBG [in mmol/L] * FINS [in units/L])/22.5. Values of **a**–**f** are mean ± SD. n = 8 rats per group. *P < 0.05 vs. normal group, *^#^P < 0.05 vs. both normal and PBS group

Furthermore, HOMA-β in the PBS group was significantly lower than in the normal group, and SHED and BMSCs injection reversed the decrease of HOMA-β in GK rats; HOMA-IR in the SHED group and BMSCs group was significantly higher than in the PBS group, and there was no significant difference between the SHED group and BMSCs group (Fig. 2).

### Effects of SHED on pancreatic islet histological changes

In the PBS group, obvious atrophy and morphologically irregular islets were observed. After calculation and statistics, the proportion of irregular islets in the SHED group, BMSCs group and PBS group was significantly higher than in the normal group, and the ratio of irregular islets in the SHED group and BMSCs group was significantly lower than the PBS group.

**Fig. 3:**
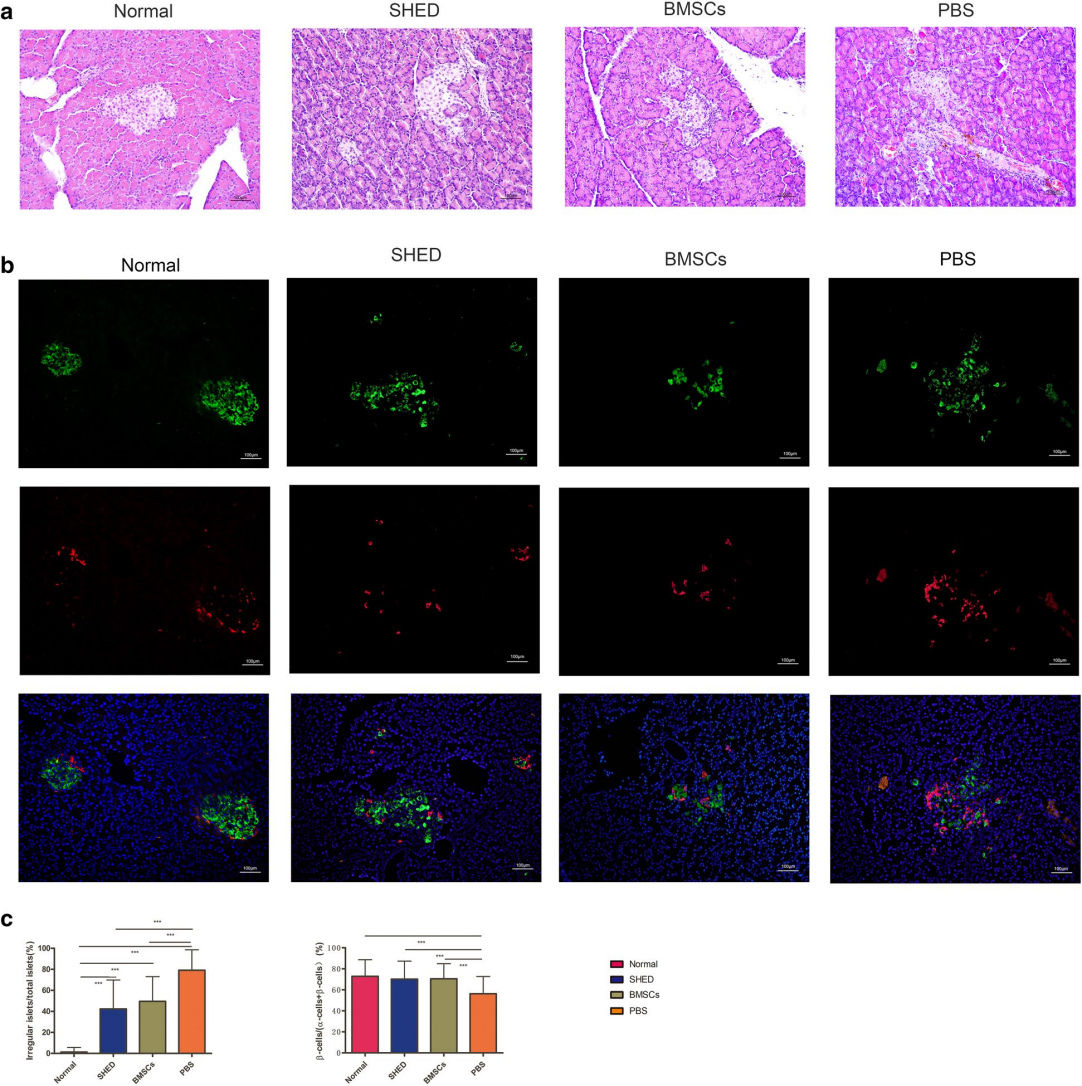
Infusion of SHED promotes restoration of pancreatic islets in T2DM rats. **a** Pancreas histology was studied via HE staining, observed under light microscopy and focused on islet structures. **b** Pancreatic islets were characterized by immunofluorescence according to the presence and distribution of insulin-producing (green) and glucagon-producing (red) cells in four groups of rats at 8 weeks after infusion. Images were composite overlays of the individually stained nuclei, insulin and glucagon. SHED and BMSCs injection significantly improved abnormalities in pancreatic islets. **c** The proportion of the irregular islets and the proportion of β-cells in 4 group. ***P<0.001

Immunofluorescence co-staining showed that the islets of normal rats had a round or elliptical shape, and islet β-cells were located in the center of the islets, which constituted most of the islets, and a small amount of scattered α-cells were distributed on the outer edges of the islets. In the PBS group, the islet structure collapsed and was disordered, and α-cells were not only located at the edge of the islets but also in the center of the islets, and the proportion of β-cells significantly decreased. Additionally, SHED and BMSCs injection significantly improved the abnormalities of the pancreatic islets (Fig. 3).

### Effects of SHED on liver histology and SHED detection in the liver

Periodic acid-Schiff staining showed the storage of glycogen in the liver. In the normal group, glycogen is a fine red particulate material that is widely present in the cytosol. In the PBS group, the amount of glycogen deposition was reduced and mottled vacuoles were observed while SHED and BMSC injection significantly improved the glycogen storage in T2DM rats.

**Fig. 4:**
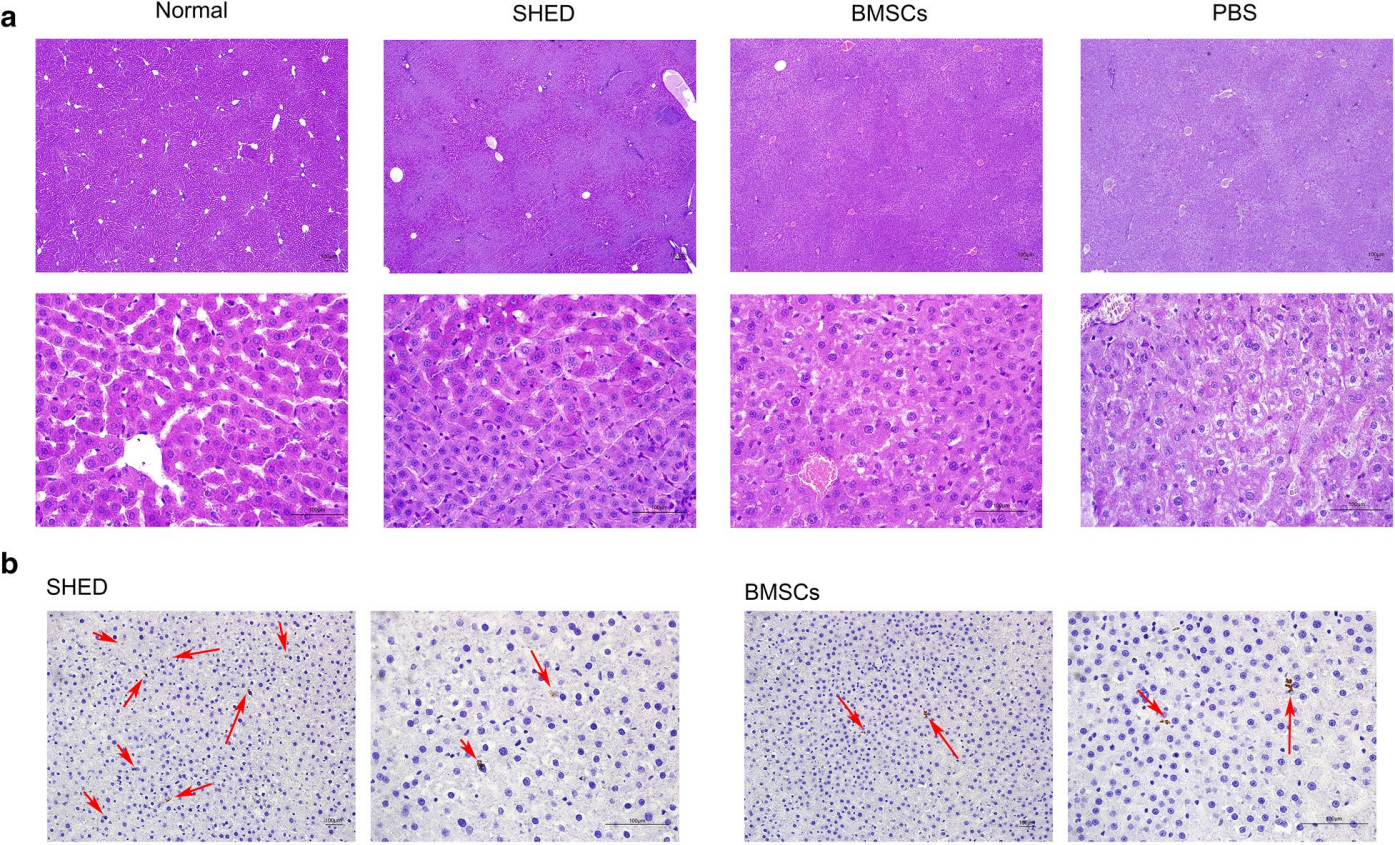
Effects of SHED on liver histology and SHED engraftment in the liver. **a** PAS staining showed the storage of glycogen in the liver. SHED and BMSC injection significantly improved glycogen storage in T2DM. **b** Immunohistochemical representative micrograph of liver tissue from the SHED group and BMSCs group. Red arrow indicated SHED and BMSCs engraftment

Moreover, human DNA PKcs were detected in the liver at 8 weeks after MSC administration. SHED and BMSCs were found in liver tissue (Fig. 4).

### Quantitative real-time PCR analysis and western blotting of the liver

Real-time PCR analysis showed that SHED and BMSCs significantly reversed the diabetic-induced increase of G-6-Pase, Pck1 and PK. In contrast, MSC injection significantly reversed the diabetic-induced decrease of GSK3b, GLUT2, and PFKL.

**Fig. 5:**
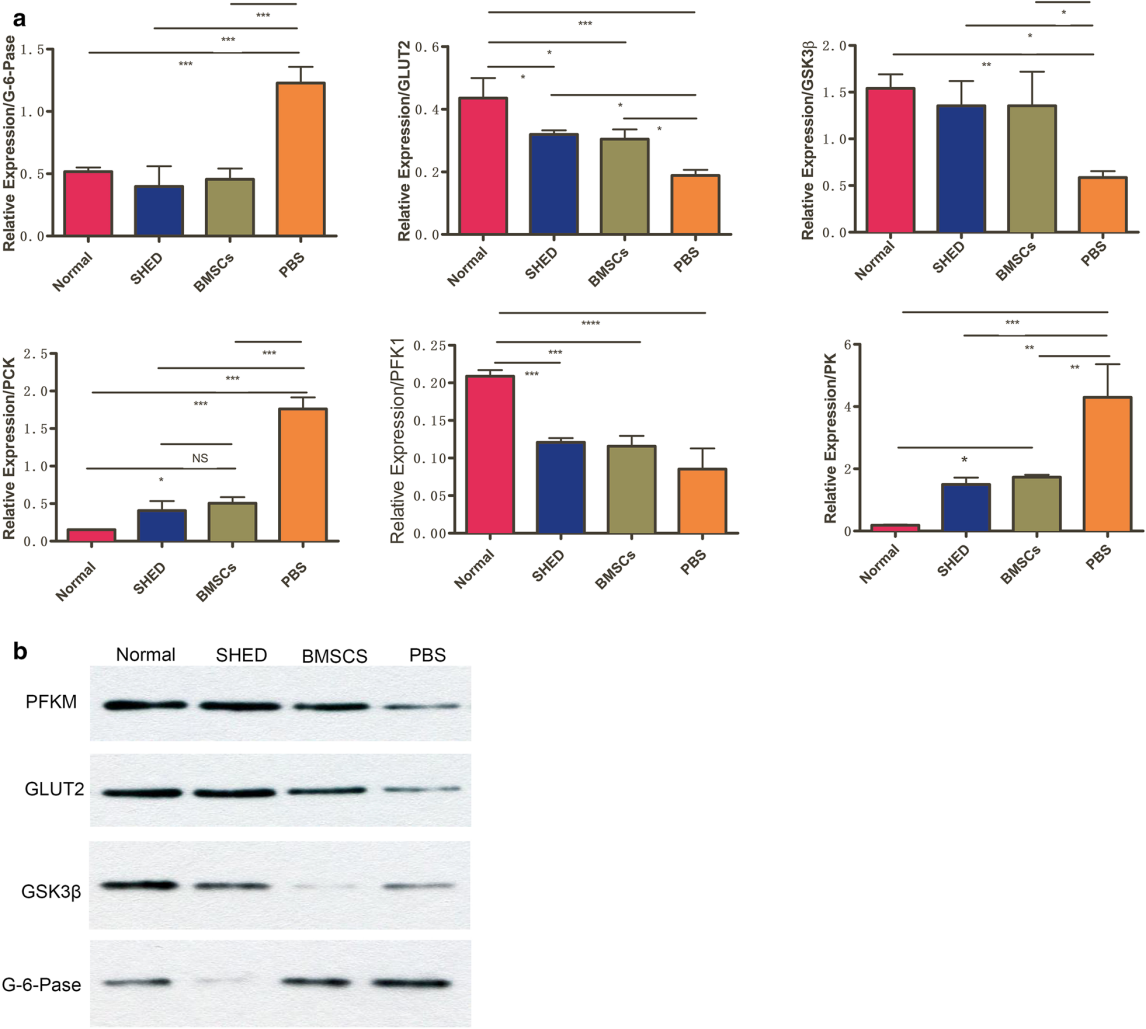
Quantitative real-time PCR analysis and western blotting of the liver. **a** Effect of SHED on G-6-Pase, Pck1, GSK3b, GLUT2, PFKL and PK mRNA expression by real-time PCR. *P < 0.05, **P < 0.01, ***P < 0.001, ****P < 0.0001. **b** Effect of MSCs on GSK3β, G-6-Pase, GLUT2 and PFKM expression by western blotting

In addition, western blotting demonstrated that SHED and BMSCs significantly reversed the diabetic-induced increase of G-6-Pase and PK; in contrast, MSCs injection significantly reversed the diabetic-induced decrease of GSK3β, GLUT2 and PFKM (Fig. 5).

## Discussion

By 2030, diabetes mellitus (DM) will be the 7th leading cause of death worldwide [1]. The World Health Organization (WHO) claims that the incidence of DM has increased to 422 million individuals [2]. DM is associated with complications that affect patient quality of life. For example, more than 50% of diabetic patients present other physiological disorders, such as cardiovascular diseases (heart attacks and strokes), higher susceptibility to infections, kidney failure, and retinopathy. T2DM is the most common type of DM, and it is characterized by insulin resistance in skeletal muscle, adipose tissue, and the liver. Defective β-cell secretory function, fasting hyperglycemia and hyperinsulinemia, and increased hepatic glucose production are also typical characteristics of T2DM [3–5].

The antidiabetic effect of MSCs, such as BMSCs, umbilical cord blood (UCB) MSCs, and adipose tissue-derived MSCs (ADSCs), has been demonstrated in different animal models and patients with T1DM [10–12] and T2DM [13, 14]. Transplantation of syngeneic or allogeneic MSCs is useful in preventing diabetes onset and also retarding its progression.

In our research, the present data clearly demonstrated that SHED administration ameliorated T2DM in GK rats, including hyperglycemia, the function and structure of pancreatic islets and insulin resistance in the liver. In addition, we found that there was no significant difference of the effects on DM between SHED and BMSCs.

Of note, at 3 weeks after injection, fasting blood glucose in the SHED group was significantly lower than in the PBS group. In the SHED group, fasting blood glucose was significantly lower after injection over 8 weeks except at 5 weeks. At 2, 3, 4, 6, and 7 weeks after injection, the non-fasting blood glucose level in the SHED group was significantly lower than in the PBS group. At 5 weeks after administration, non-fasting blood glucose levels in the BMSCs and SHED groups were significantly lower than in the PBS group, and there was no significant difference between the BMSCs group and the SHED group. Our results differ from that of previous studies, which showed that the non-fasting blood glucose level decreased significantly during the entire treatment. One possible reason was that the animal model in this research was different from STZ-induced diabetic animals in the previous studies. This study is the first time that GK rats were used to explore the effect of MSC on T2DM. GK rats, a non-obese and spontaneous (genetic) T2DM experimental model, have been widely used to investigate the development of T2DM and its complications. These animals were obtained by repeated selective breeding of glucose intolerant Wistar rats exhibiting peripheral insulin resistance and impaired insulin secretion [29, 30]. Another reason may be the differences among individual GK rats, so a study with a large sample is necessary in the future.

Regarding the effect of SHED on fasting glucose and non-fasting glucose, we supposed that SHED may influence blood glucose levels by decreasing insulin resistance in T2DM rats, so IPGTT tests were performed and the HOMA-β index and HOMA-IR index were calculated. The results suggested that SHED can ameliorate hyperglycemia by modulating β-cell function and insulin resistance.

Consistent with previous results, we found that the administration of SHED prevented pancreas damage and liver injury. Pancreatic islet histological analysis showed that MSC infusion prevented the destruction of pancreatic islets and produced morphologies similar to normal islets, which were organized with insulin-producing cells located centrally and glucagon-producing cells located peripherally. Nevertheless, SHED infusion ameliorated the ratio of insulin producing β-cells to total pancreatic islet cells. Meanwhile, the SHED injection significantly improved the glycogen storage in T2DM rat livers.

Mesenchymal stem cells may contribute to tissue recovery due to their immunomodulatory potential and paracrine mechanisms [15–17]. We suspect that this function may be related to SHED engraftment in the liver promoting glycogen synthesis, inhibition of glycolysis, increased gluconeogenesis and insulin resistance. In the present study, the immunohistochemical data confirmed the engraftment of a few SHED injected through the tail vein into the liver at 8 weeks after injection. Although the exact mechanism involved in stem cell homing to sites of injury remains elusive, hypoxia, inflammation and high glucose, all of which are present in diabetic livers, may induce the migration and proliferation of MSCs. In our experiments, no MSCs were found in islets with IHF, while MSCs were found in liver. We suspect that for the larger blood flow to the liver, MSCs injected through the tail vein are more likely to reach the liver instead of pancreatic islets. Another reason may be the chemotaxis of impaired liver and islets was different.

There is a decrease in glycogen synthesis, inhibition of glycolysis, increased gluconeogenesis and insulin resistance in the livers of T2DM rats. MSCs administration selectively acts on different key enzymes that play important roles. PAS staining showed that SHED administration significantly improved the glycogen storage in GK rat livers. Additionally, real-time PCR analysis showed that SHED and BMSCs significantly reversed the diabetic-induced increase of G-6-Pase and Pck1. In contrast, MSC injection significantly reversed the diabetic-induced decrease of GSK3b, GLUT2, PFKL and PK. In addition, western blotting demonstrated that SHED and BMSCs significantly reversed the diabetic-induced increase of G-6-Pase; in contrast, MSC injection significantly reversed the diabetic-induced decrease of GSK3β, GLUT2 and PFKM. GSK3 is one of the most important regulators of glycogen synthase, which can be phosphorylated to be inactivated. PFK is a key enzyme in the first step of catalyzing glycolysis, which is irreversible. PK catalyzes the conversion of phosphoenolpyruvate to acetone, as the last step in the process. GLUT2 is a glucose transporter expressed on the liver and islet cell membranes, and abnormal GLUT2 synthesis can lead to insulin resistance. G-6-Pase reversibly catalyzes the production of fructose-6-phosphate by glucose-6-phosphate, and PCK catalyzes the production of phosphoenolpyruvate by oxaloacetate.

## Conclusion

Stem cells from human exfoliated deciduous teeth offer a potentially effective therapeutic modality for ameliorating T2DM, including hyperglycemia, insulin resistance, pancreatic islet and liver damage, and decreased glycogen synthesis, inhibition of glycolysis and increased gluconeogenesis in the liver.

## Abbreviations

DM: diabetes mellitus
T2DM: type 2 diabetes mellitus
SHED: stem cells from human exfoliated deciduous teeth
MSCs: mesenchymal stem cells
GK rats: Goto-Kakizaki rats
BMSCs: bone marrow-derived mesenchymal stem cells
IPITTs: intraperitoneal insulin tolerance tests
IRTs: insulin release tests
HE: hematoxylin–eosin
PAS: periodic acid-Schiff
IHC: immunohistochemistry
PBS: phosphate buffer saline
α-MEM: α-modified Eagle’s medium
FBS: fetal bovine serum
MCM: mesenchymal cells condition medium
GAPDH: glyceraldehyde-3phosphate dehydrogenase
FBG: fasting blood glucose
FINS: fasting serum insulin
HOMA: homeostatic model assessment
USB-SCs: umbilical cord blood-derived mesenchymal stem cells
ADMSCs: adipose-derived mesenchymal stem cells
